# Plumbagin Ameliorates CCl_**4**_-Induced Hepatic Fibrosis in Rats via the Epidermal Growth Factor Receptor Signaling Pathway

**DOI:** 10.1155/2015/645727

**Published:** 2015-10-15

**Authors:** Si Chen, Yi Chen, Bi Chen, Yi-jing Cai, Zhuo-lin Zou, Jin-guo Wang, Zhuo Lin, Xiao-dong Wang, Li-yun Fu, Yao-ren Hu, Yong-ping Chen, Da-zhi Chen

**Affiliations:** ^1^Department of Hepatopathy, Ningbo No. 2 Hospital, Ningbo, Zhejiang 315010, China; ^2^Wenzhou Key Laboratory of Hepatology, Department of Infectious Disease, Hepatology Institute of Wenzhou Medical University, The First Affiliated Hospital of Wenzhou Medical University, Wenzhou, Zhejiang 325000, China; ^3^Department of Spine Surgery, The First Affiliated Hospital of Wenzhou Medical University, Wenzhou, Zhejiang 325000, China; ^4^Department of Urology, The First Affiliated Hospital of Wenzhou Medical University, Wenzhou, Zhejiang 325000, China; ^5^Li Ka Shing Faculty of Medicine, University of Hong Kong, Hong Kong

## Abstract

Epidermal growth factor (EGF) and its signaling molecules, EGFreceptor (EGFR) and signal transducer and activator of transcription factor 3 (STAT3), have been considered to play a role in liver fibrosis and cirrhosis. Plumbagin (PL) is an extracted component from the plant and has been used to treat different kinds of cancer. However, its role in regulation of EGFR and STAT3 during liver fibrosis has not been investigated. In this study, the effects of PL on the regulation of EGFR and STAT3 were investigated in carbon tetrachloride (CCl_4_) induced liver fibrosis and hepatic stellate cells (HSC-T6). PL significantly attenuated liver injury and fibrosis in CCl_4_ treated rats. At concentrations of 2 to 6 *μ*M, PL did not induce significant cytotoxicity of HSC-T6 cells. Moreover, PL reduced phosphorylation of EGFR and STAT3 in both fibrotic liver and heparin-binding EGF-like growth factor (HB-EGF) treated HSC-T6 cells. Furthermore, PL reduced the expression of *α*-SMA, EGFR, and STAT3 in both fibrotic liver and HB-EGF treated HSC-T6 cells. In conclusion, plumbagin could ameliorate the development of hepatic fibrosis through its downregulation of EGFR and STAT3 in the liver, especially in hepatic stellate cells.

## 1. Introduction

Liver fibrosis is a wound-healing response to chronic liver injuries associated with various etiologies including viral, metabolic, genetic, and cholestatic liver disease [1]. The underlying pathological process involves the activation of quiescent hepatic stellate cells (HSCs) into contractile myofibroblast-like cells, which secrete excessive extracellular matrix (ECM) proteins in the liver [2, 3]. Hepatic fibrosis ultimately leads to cirrhosis or hepatocellular carcinoma (HCC) and represents a serious health-care problem worldwide [4]. Sustained advances in our understanding of fibrogenesis have generated the recognition that liver fibrosis and/or even cirrhosis may be reversible; thereby there is an urgent need to develop effective antifibrotic treatments [5, 6].

A variety of animal models mimic different pathogeny (viral hepatitis, alcoholic liver disease, NAFLD, autoimmune liver diseases, cholestasis, etc.) induced liver fibrosis and cirrhosis in mice, rats, rabbits, and pigs [7]. (1) Chemical compounds, such as CCl4, thioacetamide, and dimethylnitrosamine, injure hepatocytes, trigger secondary inflammatory reaction, and induce liver fibrosis and cirrhosis [8–10]. (2) Alcoholic liver disease can be mimicked by ethanol [11] and NAFLD can be mimicked by choline-deficient, L-amino acid-defined, methionine-deficient diet and high-fat diet administrated on the animals [12–15]. (3) Immunogens including plant protein concanavalin A and xenogeneic serum cause allergic reaction and inflammation, stimulating fibrosis formation [16, 17]. Those approaches mimic the process of autoimmune liver diseases provoking liver fibrosis. (4) An operation of bile duct ligation is used as the animal model of cholestasis [18]. (5) Genetic overexpression of TGF-*β*1 gene has been shown to induce cirrhosis in mice [19]. Because of the simple operation and stable affection, intraperitoneal infection of CCl_4_ was used as the approach of our mice model.

Recent studies have demonstrated that epidermal growth factor receptor (EGFR) plays a critical role in both fibrosis and HCC [20, 21]. Being a transmembrane protein endowed with tyrosine kinase activity, EGFR binding with its ligands leads to dimerization and phosphorylation of tyrosine residues in its cytosolic domains. The phosphorylated tyrosine residues function as a kinase to activate intracellular signaling molecules such as extracellular-regulated kinase (ERK) and signal transducer and activator of transcription 3 (STAT3) [22–24]. As a membrane protein, EGFR can activate intracellular signaling molecule STAT3, which functions as a transcription factor to carry EGFR signal to the nuclear and regulate target gene expression. Recent studies demonstrated that overexpression of the ligands of EGFR result in fibrosis in the pancreas, kidney, and hypertrophic scars indicating the connection between fibrosis and epithelial metaplasia [25, 26]. Moreover, pharmacological inhibition of EGFR signaling could effectively regress fibrosis in some animals [20], and inhibition of EGFR might have therapeutic potential for fibrotic kidney disease [27].

Plumbagin (5-hydroxy-2-methyl-1, 4-naphthoquinone, and PL) is an active constituent extracted from the roots of traditional medicinal plant* Plumbago zeylanica* L. (Plumbaginaceae). PL has been demonstrated to have various biological activities, such as the induction of apoptosis, anti-inflammation, antiangiogenesis, anticancer, and antioxidant activity [28–30]. PL has been shown to inhibit LPS-induced I*κ*B*α* degradation and activation of NF-*κ*B and ERK in liver [31]. Moreover, PL ameliorated experimental autoimmune encephalomyelitis (EAE) through downregulation of JAK-STAT and NF-*κ*B signaling pathways [32]. However, the application of PL for the treatment of liver fibrosis has not been evaluated in liver fibrosis model, especially the regulation of EGFR/STAT3 pathway in fibrotic liver. Therefore, the current study investigated the effect of PL on liver fibrosis in a rat fibrotic liver model as well as in HSC-T6 cells and explored the possible underlying mechanisms of liver fibrosis, particularly the EGFR/STAT3 pathway.

## 2. Materials and Methods

### 2.1. Animals and Treatments

**Figure 1:**
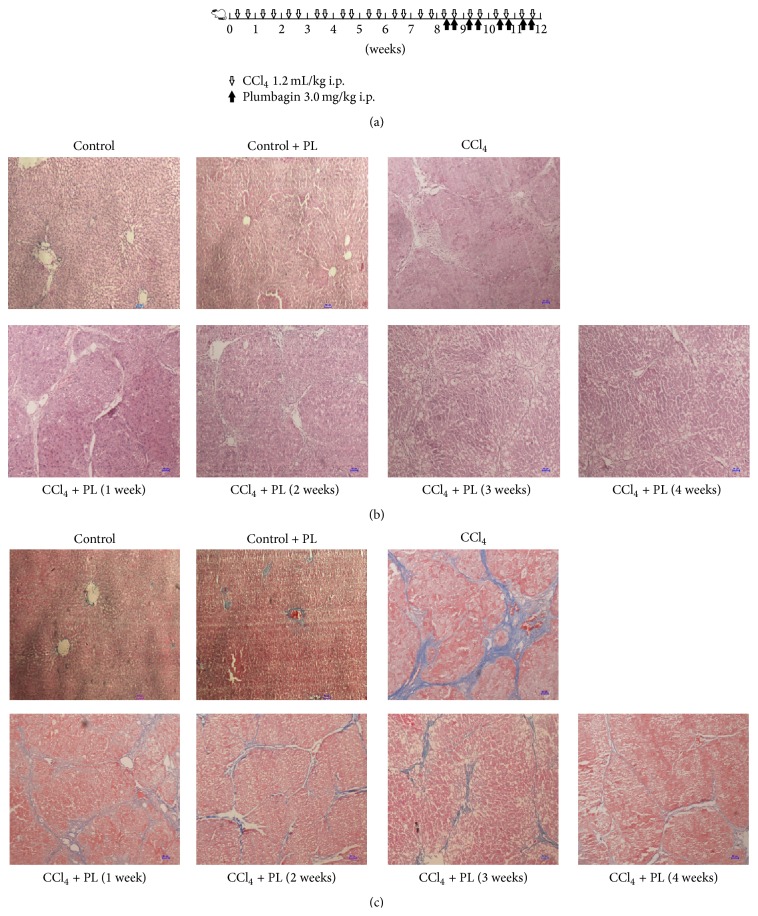
Schedule of CCl_4_ administration and histology of liver injury and fibrosis in rats. (a) shows the schedule of intraperitoneal (i.p.) injection of CCl_4_. (b) displays that liver sections were stained with hematoxylin and eosin (HE) in rats treated with vehicle, vehicle + PL, CCl_4_, and CCl_4_ + PL. (c) represents liver tissues with Masson's trichrome staining in rats treated with vehicle, vehicle + PL, CCl_4_, and CCl_4_ + PL (Magnification: ×100, scale bar = 50 *μ*m).

Forty-two male Sprague-Dawley rats (250–300 g) were used for* in vivo* experiments. The rats were obtained from the Shanghai Laboratory Animal Center (Shanghai, China) and had free access to standard laboratory water and chow. All the animals were randomly divided into four groups: control (*n* = 6), carbon tetrachloride (CCl_4_) (*n* = 6), CCl_4_ plus PL treatment (*n* = 24), and control plus PL treatment (*n* = 6). The CCl_4_ plus PL treatment group was further divided into four subgroups according to different time points: 1 week (*n* = 6), 2 weeks (*n* = 6), 3 weeks (*n* = 6), and 4 weeks (*n* = 6). Liver fibrosis was induced in the CCl_4_ and the CCl_4_ plus PL treatment group by intraperitoneal (i.p.) injection of CCl_4_ (Sigma, St. Louis, MO, USA) (dissolved CCl_4_ at 40% in olive oil) at 2.5 mL/kg body weight twice a week for 12 weeks [33]. The rats in the CCl_4_ plus PL treatment group were injected with plumbagin (Sigma, St. Louis, MO, USA) (dissolved PL in DMSO to 20 mg/mL storing at 4°C and diluted in olive oil to 2 mg/mL when used) at 3.0 mg/kg body weight twice a week intraperitoneally, starting at the 8th week after the first administration of CCl_4_ and lasting for 1, 2, 3, and 4 weeks, respectively (shown in Figure 1). The animals in the control and the control plus PL treatment groups were treated similarly including i.p. injection with the same volume of olive oil and the same volume of vehicle with or without PL for 4 weeks, respectively. Rats were sacrificed at the end of 12 weeks or other time points as indicated. Blood and liver tissue samples were collected for further study. The above study was in accordance with the ethical standards of the institutional animal committee of Wenzhou Medical University.

### 2.2. Analysis of Serum Transaminase Activity

When the animals were anesthetized with 10% chloral hydrate solution (3.5 mL/kg, i.p. injection), the blood samples from each group were collected and centrifuged at 4000 rpm at 4°C for 10 min. Serum levels of alanine aminotransferase (ALT) and aspartate aminotransferase (AST) were determined using an automatic biochemistry analyzer (Abbott Laboratories, USA).

### 2.3. Histological Examination of Liver Injury

A small portion of the liver was fixed in 10% formalin for 12 h and then paraffin-embedded. All the tissue specimens were cut into 4 *μ*m sections. The changes in liver histology were examined using hematoxylin and eosin (HE) and Masson's trichrome staining, which were observed under a light microscope. Three sections per animal liver were blindly examined by an experienced liver pathologist. Fibrosis in the Masson's trichrome staining sections was evaluated according to the Semiquantitative Scoring System [34]. The remaining liver was washed with ice-cold saline and cut into pieces that were immediately stored at −80°C for further analysis.

### 2.4. Cell Culture and Analysis of Cell Viability (MTT Assay)

HSC-T6 was purchased from XiangYa Central Experiment Laboratory (Changsha, Hunan province, China). The HSC-T6 cells were maintained in Dulbecco's modified Eagle's medium (DMEM) supplemented with 10% fetal bovine serum (FBS) and 1% penicillin and streptomycin in an incubator of 5% CO_2_ at 37°C.

The colorimetric MTT (3-[4,5-dimethylthiazol-2-yl]-2,5-diphenyl-tetrazolium bromide) assay was used to evaluate the cytotoxicity of plumbagin. Briefly, cells were seeded in a 96-well plate at a density of 1 × 10^4^ cells/mL for 24 h. Cells were treated with plumbagin at different concentrations (0, 2, 4, 6, 8, 10, 12, 14, and 16 *μ*M) for 24 h. Each concentration was repeated six times. After the incubation period, cells were replaced with 200 *μ*L fresh medium and 20 *μ*L of the MTT solution (5 mg MTT per mL PBS) was added to each well. The microplate was incubated for another 4 h at 37°C in a humidified atmosphere (5% CO_2_). The supernatant was removed and 150 *μ*L dimethyl sulfoxide (DMSO) was added to each well to solubilize the crystals. The spectrophotometrical absorbance of the samples was measured using a microplate (ELISA) reader at a wavelength of 550 nm. The percentage of viable cells was estimated by comparing with untreated control cells.

### 2.5. Treatment of HSC-T6 Cells

Recombinant human HB-EGF (PeproTech, Rocky Hill, NJ, USA) was dissolved in sterile distilled water. AG1478 (Sigma, St. Louis, MO, USA) were dissolved in 0.1% DMSO. Plumbagin (Sigma, St. Louis, MO, USA) was dissolved in DMSO to 0.1 M and stored at −20°C. Subsequent dilutions were made in cell culture medium. After 24 h culture in six-well plates at a density of 1.5 × 10^5^ cells per well, HSC-T6 cells were separated into seven groups: (A) medium control: 24 h incubation with 10% FBS in DMEM; (B) DMSO control: same as medium control except addition of 0.1% DMSO; (C) HB-EGF (80 ng/mL, dissolved in DMEM): cells were incubated with HB-EGF for 24 h, (D) HB-EGF + AG1478 (5 *μ*M), (E) HB-EGF + PL (2 *μ*M), (F) HB-EGF + PL (4 *μ*M), and (G) HB-EGF + PL (6 *μ*M). The total RNA and cellular proteins were extracted for further analysis.

### 2.6. RNA Isolation and Quantitative Real-Time RT-PCR

Total RNA was extracted from liver and cell samples using EASYspin Plus RNA kit (Aidlab Biotechnologies Co., China) according to the manufacturer's protocol. The cDNA was carried out using 2x Power Taq PCR MasterMix (BioTeke, China). The cDNA was subjected to quantitative real-time RT-PCR (qPCR) using SYBR premix Ex Taq (TaKaRa, Dalian, China) with the ABI 7300 Sequence Detection System (Applied Biosystems, Foster, CA). Primer sequences (forward and reverse) are as follows: *α*-SMA, GCTCTGTAAGGCGGGCTTTG and ACGAAGGAATAGCCACGCTCA; EGFR, CATCCAGTGCCATCCAGAAT, and CTTCCAGACCAGGGTGTTGT; STAT3, AACGACCTGCAGCAATACCA, and TCCATGTCAAACGTGAGCGA; *β*-actin, CACCCGCGAGTACAACCTTC, and CCCATACCCACCATCACACC. The relative expression of each gene was normalized to the expression levels of *β*-actin and the results were analyzed by the comparative cycle threshold method.

### 2.7. Protein Isolation and Western Blot Analysis

Liver homogenates and cells were lysed, and the cellular protein was isolated as previously described [35]. Twenty micrograms of proteins from each sample was mixed with 4x gel loading buffer and boiled at 100°C for 5 min. The samples were loaded to 10% polyacrylamide gel (SDS-PAGE). After electrophoresis, gels were electrotransferred onto nitrocellulose membranes, which were then blocked by 5% skimmed milk for 2 h at room temperature. The primary antibodies against EGFR (1 : 1000; Cell Signaling, Danvers, MA, USA, 3265s), p-EGFR (1 : 1000; Cell Signaling, Danvers, MA, USA, 3777p), STAT3 (1 : 1000; Cell Signaling, Danvers, MA, USA, 4904p), p-STAT3 (1 : 1000; Cell Signaling, Danvers, MA, USA, 9145p), *α*-SMA (1 : 200; Boster, Shanghai, China, BM0002), and GAPDH (1 : 1000; Santa Cruz, Dallas, Texas, USA, SC-51907) were incubated with the membranes in 1x TBS/T (1x Tris Buffered Saline containing 0.1% Tween-20) plus 3% skim milk overnight at 4°C, respectively. After being washed with 1x TBS/T four times for 6 min each, the membranes were incubated with a secondary horseradish peroxidase (HRP) at room temperature for 60 min. The membranes were then washed again 4 times for 6 min each in 1x TBS/T. The protein bands were visualized using a chemiluminescent HRP substrate (Millipore Corporation, Billerica, USA) and exposed to Kodak film.

### 2.8. Statistics

Data are expressed as mean ± S.D. of independent experiments. Statistical significance of difference was determined by a one-way analysis of variance (ANOVA) followed by the least significant difference (LSD) test. A significant value was defined as *P* < 0.05.

## 3. Results

### 3.1. Plumbagin Attenuated CCl_4_-Induced Liver Injury in Rats

**Figure 2:**
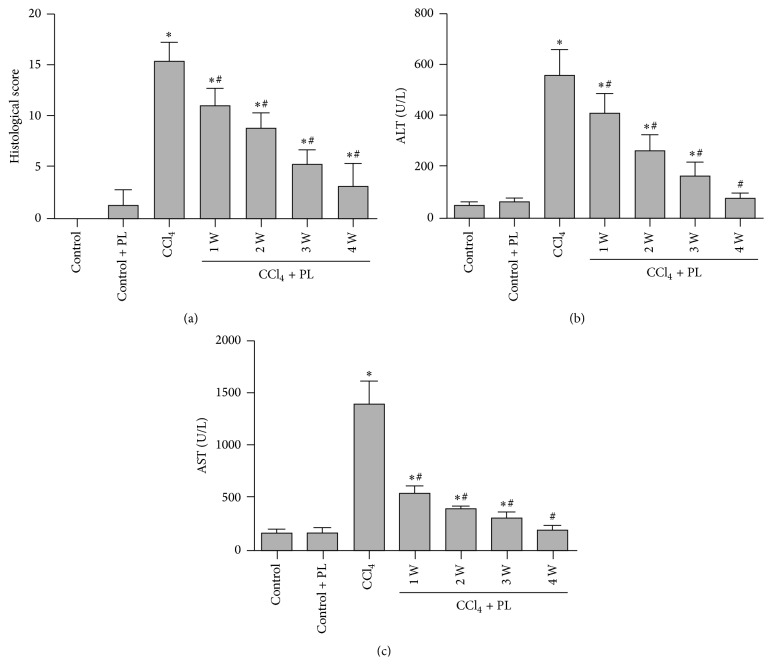
Effect of PL on liver fibrosis and liver enzymes in rats from different groups. (a) represents liver fibrosis score from Masson's trichrome staining according to the Semiquantitative Scoring System. (b) and (c) show the levels of ALT and AST from different groups, respectively. *∗* indicates *P* < 0.05 compared to control group while # indicates *P* < 0.05 compared to CCl_4_ group.

Histological examination using HE and Masson's trichrome staining is shown in Figure 1. After HE staining, livers from the control and control plus PL group showed normal lobular architecture with central veins and radiating hepatic cords (Figure 1(b)). The CCl_4_-treated group showed extensive disruption of liver architecture, including hepatocellular necrosis, inflammatory cell infiltration, and fibrosis (Figure 1(b)). In the liver of rats treated with PL, damage was reduced, especially in the rats treated for 4 weeks (Figure 1(b)). Masson's trichrome staining showed that few collagen fibers were presented in the liver tissues of the control and control plus PL-treated rats (Figure 1(c)). There was large amount of total collagen deposition in the liver of CCl_4_-treated rats that presents as the formation of pseudolobules (Figure 1(c)). However, PL treatment reduced the content of collagen fibers in the liver (Figure 1(c)). In addition, when fibrosis score was evaluated in liver sections by Semiquantitative Scoring System (Figure 2(a)), significantly higher histological fibrosis score was found in the CCl_4_ group compared to control group (*P* < 0.05). After treatment with PL, the score was significantly reduced, especially in the liver of 4-week treatment group (*P* < 0.05). Serum levels of ALT and AST in the CCl_4_-treated rats were significantly higher than those in control rats. The abnormal levels of these liver enzymes were significantly reduced with the PL treatment in a time-dependent manner (*P* < 0.05) as shown in Figures 2(b) and 2(c).

**Figure 3:**
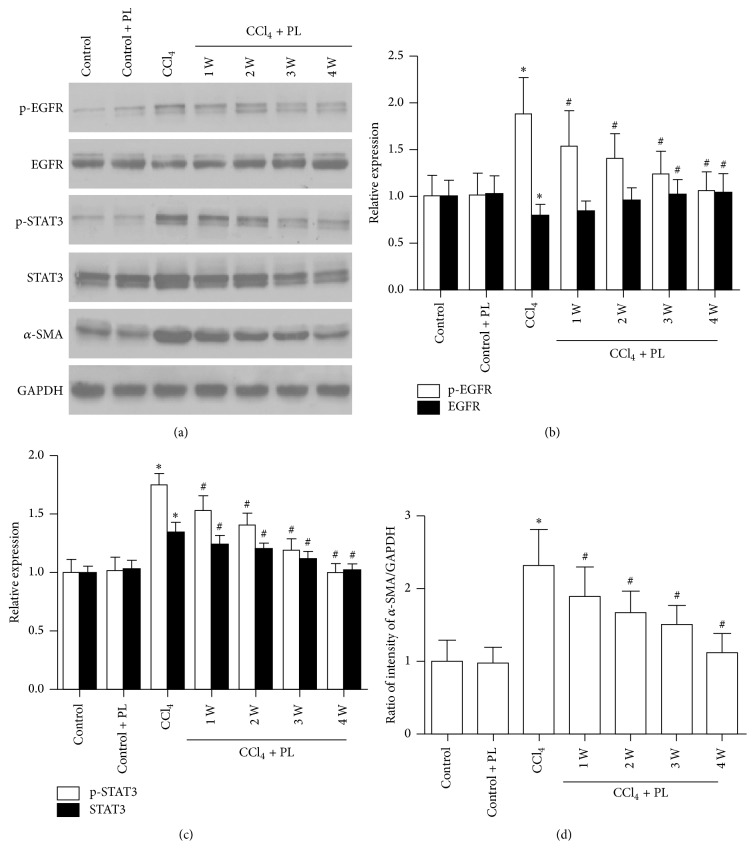
Plumbagin regulation of protein levels of *α*-SMA, EGFR, and STAT3 in the liver of rats from different groups. (a) represents typical picture of Western blot *α*-SMA, EGFR, p-EGFR, STAT3, and p-STAT3 in the liver. (b) shows histogram of EGFR and p-EGFR densities from the bands; (c) displays histogram of STAT3 and p-STAT3 densities from the bands; and (d) shows histogram of *α*-SMA densities. Relative levels of these proteins were normalized to the level of GAPDH. Data represent the mean ± SD from 6 mice. *∗* indicates *P* < 0.05 compared to control group while # indicates *P* < 0.05 compared to CCl_4_ group.

**Figure 4:**
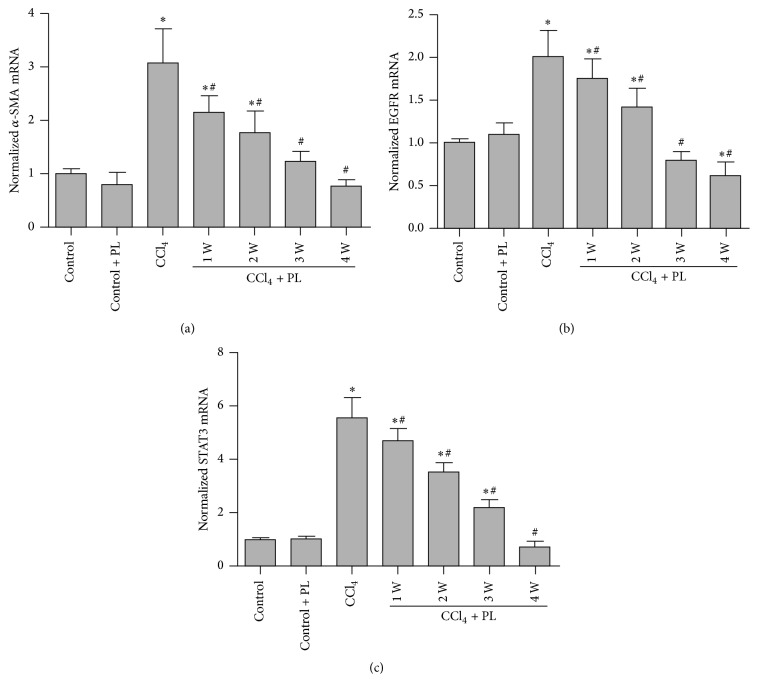
Plumbagin regulation of mRNA levels of *α*-SMA, EGFR, and STAT3 in the liver from different groups. (a) shows the mRNA level of *α*-SMA; (b) represents the mRNA level of EGFR; and (c) shows the mRNA level of STAT3 in the liver. Data represent the mean ± SD of 6 mice and relative expression was normalized to the level of beta-actin. *∗* indicates *P* < 0.05 compared to control group while # indicates *P* < 0.05 compared to CCl_4_ group.

To test whether PL affected stellate cell activation, the expression of *α*-SMA, myofibroblast markers, in the liver was examined. As shown in Figures 3 and 4, *α*-SMA mRNA and protein were increased in the CCl_4_-treated rats, while a significant reduction of *α*-SMA mRNA and protein was observed in the liver of PL-treated rats, especially the 4-week treatment group (*P* < 0.05).

### 3.2. Plumbagin Inhibited the Activation of EGFR and STAT3 in CCl_4_-Treated Rats

As shown in Figure 3, although there were some changes of total EGFR and total STAT3 after CCl_4_ treatment, significantly increased levels of p-EGFR and p-STAT3 were observed in the CCl_4_ group compared with the control group (*P* < 0.05). Moreover, significant reduction of p-EGFR and p-STAT3 was observed after 3 and 4 weeks of treatment with PL. In addition, the mRNA levels of EGFR and STAT3 were increased in the CCl_4_-treated group compared to control. The treatment with PL significantly reduced the elevations (*P* < 0.05) (Figure 4).

### 3.3. Cytotoxicity of Plumbagin to HSC-T6 Cells

**Figure 5:**
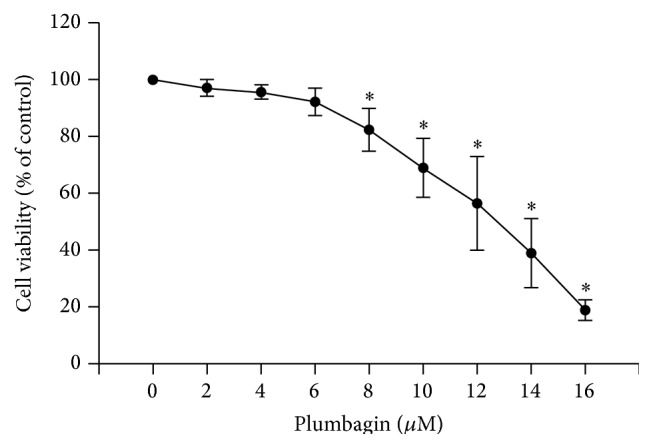
Cytotoxicity of plumbagin to HSC-T6 cells. HSC-T6 cells were incubated with different concentrations (0, 2, 4, 6, 8, 10, 12, 14, and 16 *μ*M) of PL for 24 h. Cytotoxicity was evaluated by MTT assay. Data represent the mean ± SD of 6 independent experiments. *∗* indicates *P* < 0.05 compared with the 0 *μ*M concentration.

Cytotoxicity of PL to HSC-T6 cells was examined by MTT assay. As shown in Figure 5, plumbagin induced a dose-dependent cytotoxicity on HSC-T6 cells. Although there was no significant toxicity to the cells at the concentrations of 2, 4, and 6 *μ*M compared to 0 *μ*M concentration, significant cytotoxicity was observed at the concentrations of plumbagin higher than 8 *μ*M. Thus, the concentrations of 2, 4, and 6 *μ*M of plumbagin were used for all subsequent experiments.

### 3.4. Effect of Plumbagin on *α*-SMA, EGFR, and STAT3 in HSC-T6

**Figure 6:**
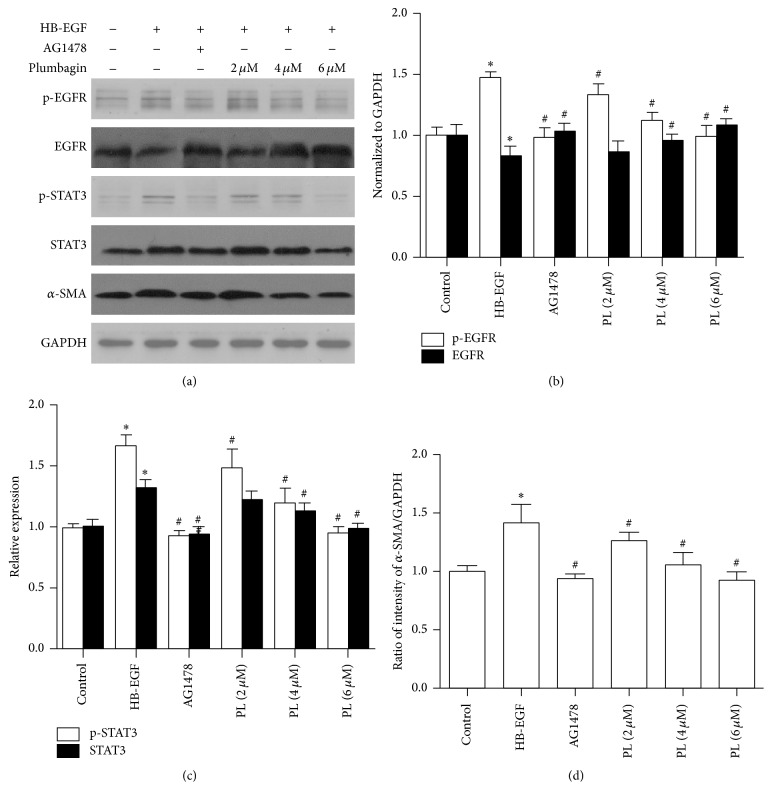
Plumbagin regulation of protein levels of *α*-SMA, EGFR, and STAT3 in HSC-T6 cells. (a) represents typical picture of Western blot *α*-SMA, EGFR, p-EGFR, STAT3, and p-STAT3 in HSC-T6 cells. (b) shows histogram of EGFR and p-EGFR densities from the bands; (c) displays histogram of STAT3 and p-STAT3 densities from the bands; and (d) shows histogram of *α*-SMA densities. Relative levels of these proteins were normalized to the level of GAPDH. Data represent the mean ± SD from 6 experiments. *∗* indicates *P* < 0.05 compared to control group while # indicates *P* < 0.05 compared to HB-EGF treatment.

**Figure 7:**
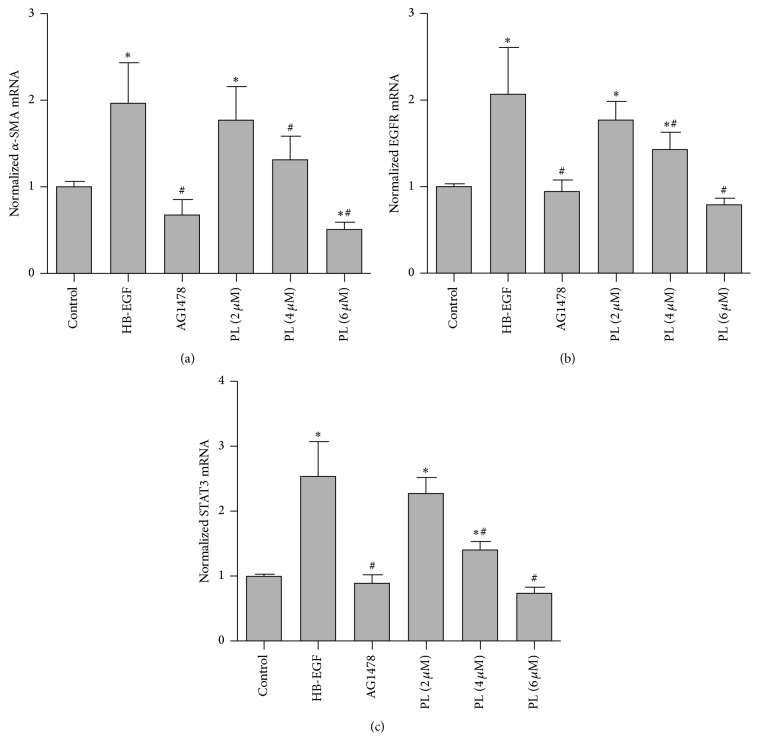
Plumbagin regulation of mRNA levels of *α*-SMA, EGFR, and STAT3 in HSC-T6 cells. (a) shows the mRNA level of *α*-SMA; (b) represents the mRNA level of EGFR; and (c) shows the mRNA level of STAT3 in HSC-T6 cells. Data represent the mean ± SD of 6 experiments and relative expression was normalized to the level of beta-actin. *∗* indicates *P* < 0.05 compared to control group while # indicates *P* < 0.05 compared to HB-EGF group.

HSC-T6 cells were further employed to investigate the effects of PL on *α*-SMA, EGFR, and STAT3. As shown in Figures 6 and 7, HB-EGF treatment induced an increase in both *α*-SMA mRNA and protein levels, while cotreatment with HB-EGF and PL significantly reduced the levels of *α*-SMA. A similar regulation of *α*-SMA was observed after cells were treated with an EGFR kinase inhibitor, AG1478. In addition, HB-EGF significantly increased the levels of p-EGFR and p-STAT3, while PL reduced the HB-EGF induced levels of p-EGFR and p-STAT3 in HSC-T6 cells, especially at the concentration of 6 *μ*M (*P* < 0.05). In addition, EGFR and STAT3 mRNA levels were increased by HB-EGF and reduced after PL treatment. Similar results from p-EGFR and p-STAT3 protein as well as mRNA levels of EGFR and STAT3 were observed in the cells treated with HB-EGF and AG1478.

## 4. Discussion

Hepatic fibrosis and cirrhosis are a major health burden affecting millions of people worldwide. In chronic inflammation, normal quiescent HSCs and perivascular fibroblasts undergo activation and transdifferentiation into myofibroblasts, which are recognized as the major cells producing hepatic ECM [36]; HSCs play a critical role in the initiation, maintenance, and progression of liver fibrosis [37, 38]. Therefore, termination of proliferation or enhancement of apoptosis of activated HSCs has been proposed as an innovative therapy for patients with chronic liver injury and fibrosis. Based on the method and doses of Jiang et al. report [33], we established liver fibrosis model by intraperitoneal infection of CCl_4_ in rats, as proved by histological evaluation and increases of the plasma aminotransferases levels as well as the upregulated expression of *α*-SMA, a typical marker of activated HSCs during the fibrotic process. Fibrosis was reduced by the treatment with PL, particularly with the 4 weeks of administration.

Emerging evidence supports the role of EGFR in fibrogenesis. The expression of EGFR was greater in hypertrophic scars than in normal skin [26]. In mice cardiac hypertrophy, the apoptosis and fibrosis of cardiac myocytes were attenuated when the EGFR transactivation was diminished [39]. STAT3 is a member of the signal transducer and activator of transcription protein family that regulates cell fate determination, renewal, differentiation, and apoptosis of various cell types, especially during embryonic developing stages [40]. Accumulating evidence indicated that blockage of EGFR resulted in dephosphorylation of STAT3 which controls its nuclear translocation [27, 41]. Xu et al. [42] showed that STAT3 signaling activation cross-talked with TGF-*β*1 signaling, which exacerbated liver injury and fibrosis. Moreover, TGF-*β*1 expression could be suppressed by siRNA-mediated knockdown of STAT3 mRNA or Janus kinase 2 inhibitor (AG490) both* in vivo* and* in vitro* [42]. Additionally, constitutive activation of STAT3 in cancer cells led to more malignant cancer phenotypes, including growth, epithelial-mesenchymal transition, migration, invasion, and metastasis. Moreover, STAT3 plays a significant role in tumor survival and therapeutic resistance [43]. Therefore, interventions to reduce activated HSCs by targeting EGFR signaling pathway may be an attractive therapeutic strategy in both hepatic fibrosis and hepatocellular carcinoma.

Our study demonstrated that the levels of p-EGFR and p-STAT3 were increased in the CCl_4_-induced rats as well as the HSC-T6 cells cultured with HB-EGF, whereas the expression of total EGFR was decreased. These results are consistent with ligand-mediated receptor endocytosis that occurs following activation of the pathway. Ligand binding to EGFR accelerates its internalization through clathrin-coated pits, which is followed by the efficient lysosomal targeting of internalized receptors resulting in receptor downregulation [44]. In order to understand the effect of PL on liver fibrosis, three concentrations of PL were chosen for the* in vitro* study. We observed that PL reduced HSC-T6 cells survival in a concentration-dependent manner. Moreover, the ratio of p-EGFR/EGFR and p-STAT3/STAT3 was decreased in the PL-treated group compared to the HB-EGF treated group in the Western bolt analysis. Consistent with* in vitro* study, the* in vivo* study showed that PL significantly alleviated CCl_4_-induced hepatic fibrosis in rats, including attenuation of liver damage as well as decreased expression of myofibroblast marker, *α*-SMA. In addition, the expression of p-EGFR/EGFR and p-STAT3/STAT3 was declined. All these results indicated that PL might alleviate CCl_4_-induced hepatic fibrosis via the EGFR/STAT3 signaling pathway.

HB-EGF is a member of the EGF superfamily and may have conflicting roles in liver fibrogenesis. Kiso et al. documented that hepatocytes produced HB-EGF in fibrotic rat livers and it may transform hepatocytes to a neoplastic phenotype [45]. Moreover, HB-EGF could promote cell proliferation in both activated primary rat HSCs and two HSC cell lines (human LX2 and rat T6) and block signal pathway that lead to HSC activation. These information were in accordance with our study [46]. However, Huang et al. demonstrated that HB-EGF knockout mice increased susceptibility to chronic thioacetamide-induced hepatic fibrosis. Both endogenous and exogenous administration of HB-EGF inhibited HSC activation in primary culture HSCs isolated from HB-EGF-null mice [47]. Similar results were demonstrated in bile duct ligation-induced liver fibrosis in these mice [48]. Although the effect of HB-EGF in the progress of liver fibrosis is variable, our* in vitro* study with HSC-T6 cells indicates that HB-EGF could promote activation of HSCs.

PL has been demonstrated to downregulate Wnt signaling in human colorectal cancer cells [49] and interrupt the pathways of NOX4 signaling in tubulointerstitial fibrosis present in diabetic nephropathy [50]. Thus, it is possible that PL could regulate hepatic fibrosis by regulating several intracellular signaling pathways related to HSCs proliferation and apoptosis. To further elucidate the underlying mechanism of PL in the treatment of liver fibrosis, investigation into several intracellular signaling pathways such as TGF*β*1-Smad3, MAPK, and PI3-Akt pathways to see whether they interact with the EGFR-STAT3 signaling is required. In addition, preliminary experiment with high doses of PL induced more HSC-T6 cell death, indicating a presence of toxicity at high dose of PL, which may cause liver injury when used in* in vivo* studies. Furthermore the concentration of PL is shown to be more toxic to other cancer cell lines and abnormal proliferated cells than to normal human cell. In our studies, PL dose more than 6 *μ*M inhibited rate of HSC-T6. Similarly, Wei [51] displays doses of both 2 *μ*M and 8 *μ*M decreasing HSC-LX2 growth, which is lower than cytotoxicity dose (8 *μ*M) on human normal embryonic hepatocyte L-O_2_. 0.5 or 1 *μ*M has no cell toxicity on HK-2 cells, an immortalized human kidney proximal tubule cell line, while 1 *μ*M of PL has antioxidant activities in diabetic nephropathy [50]. Moreover, human colorectal cancer cells, SW620 and HCT116, are inhibited by 6 *μ*M and 10 *μ*M, respectively [49]. MCF-7 cell, breast adenocarcinoma, found to be more effective in inhibiting the growth at 10 *μ*M as compared to BJ cells (normal skin fibroblasts), were less cytotoxic at the same concentration [52]. Indeed, dose of PL in anticancer activity is higher than that in impact of antioxidant and antifibrosis. However, the beneficial effects of PL in the CCl_4_-induced liver fibrosis rats and no inflammation or necrosis in “PL only” control group suggested that cytotoxic effect of PL at the concentration used was present. Moreover, the dose of PL we gave to the rats (3 mg/kg of body weight twice a week) was less than that used for the treatment of cancer, such as 2 mg/kg of body weight daily in leukemia [53] and 4 mg/kg of body weight daily in liver cancer [54].

## 5. Conclusions

Reduction of EGFR activation decreased STAT3 activation, which could attenuate the development of fibrotic progress. Moreover, plumbagin may prevent hepatic fibrosis through inhibition of EGFR phosphorylation and STAT3 activation.

## Highlights

- The administration of plumbagin was effective to the CCl_4_-induced fibrosis in rats.
- The expression of p-EGFR/EGFR and p-STAT3/STAT3 in the rat liver and HSC-T6 was increased in the CCl_4_ and HB-EGF groups.
- The expression of p-EGFR/EGFR and p-STAT3/STAT3 was downregulated by plumbagin intervention.
